# STATE VARIATION IN CANNABIS REGULATIONS CONCERNING OLDER AMERICANS

**DOI:** 10.1093/geroni/igad104.0038

**Published:** 2023-12-21

**Authors:** Fadi Martinos

**Affiliations:** University of Iowa, Iowa City, Iowa, United States

## Abstract

As of 2022, 21 states have fully legalized cannabis, 23 legalize for medical use only, and 6 states prohibit cannabis use entirely. While previous research has associated discrete aspects of state cannabis regulation with individual outcomes, little is known about the administrative rules most relevant to older persons. We previously have observed how the progressive approach to legalization across the United States, which includes flexible regulation on legal access, medical program eligibility and qualifying conditions, potency limits, care giver autonomy and others has corresponded with increased accessibility and use of cannabis among Americans over 65 years old. In this study, we source data on seven distinct state regulations pertaining to older persons and measured each on a scale reflecting permissiveness relevant to access and use. We used individual item measures to construct aggregated scores of state policies and charted changes within and among these seven state policies from 2016 to 2022. This research advances scientific understanding by capturing finite distinctions among state regulatory approaches most likely to impact older persons and offering reliable time-varying measures to be included in multi-level model formulations.

